# Multicentre Collaborative Prospective Cohort Study Investigating the Impact of Enhanced Recovery After Surgery on Kidney Transplant Outcomes: The CRAFT Study

**DOI:** 10.3389/ti.2025.15541

**Published:** 2026-01-14

**Authors:** Ruth Owen, Georgios Kourounis, Bishow Karki, Katie Connor, Charlotte Brown, Kayani Kayani, Mohamed Elzawahry, Ruth Blanco, Davide Schilirò, Paul Smith, Jenny Mehew, Miriam Manook, Carrie Scuffell, Aimen Amer, Samuel Tingle, Emily R. Thompson, CRAFT Study Collaborators

**Affiliations:** 1 Herrick Society CRAFT Study Committee, Newcastle Upon Tyne, England; 2 Institute of Transplantation, Freeman Hospital, Newcastle Upon Tyne, United Kingdom; 3 Department of Surgery, Royal Hallamshire Hospital, Sheffield, United Kingdom; 4 Department of Surgery, Edinburgh Royal Infirmary, Edinburgh, United Kingdom; 5 Department of Surgery, University Hospital of Wales, Cardiff, United Kingdom; 6 Department of Surgery, Queen Elizabeth Hospitals NHS Foundation Trust, Birmingham, United Kingdom; 7 Nuffield Department of Surgical Sciences, Oxford University Hospitals NHS Foundation Trust, Oxford, United Kingdom; 8 Department of Surgery, Leeds Teaching Hospitals, Leeds, United Kingdom; 9 Department of Surgery, Duke University, Durham, NC, United States; 10 Statistics and Clinical Research, NHS Blood and Transplant, Bristol, United Kingdom; 11 Addenbrookes Hospital, Cambridge, United Kingdom

**Keywords:** enhanced recovery after surgery (ERAS), ERAS in kidney transplantation, kidney, perioperative care, prehabilitation

## Abstract

Perioperative complications are common in kidney transplantation. Enhanced recovery after surgery (ERAS) is a well-established multimodal perioperative care pathway designed to improve patient outcomes, however, its efficacy in renal transplant remains poorly described. Participating centres included adult renal transplant recipients and 30-day follow-up data. The primary outcome was LOS. Multivariable hierarchical models compared cohorts. 213 patients were included in the study period. 18/23 UK kidney transplant centres were represented. Analysis of the perioperative care delivery demonstrated similar patterns irrespective of reported protocols, with a tendency towards ERAS-type care. Between cohorts, the incidence of complications were similar; formal ERAS 14.3%, ERAS informal 17.0%, no ERAS 12.6%; p = 0.64. Median LOS was also similar; formal ERAS 6.0 days (5.0–11.5), informal ERAS 7.0 days (5.0–10.5) vs. no ERAS 6.0 days (5.0–10.5); p = 0.75. Readmissions were comparable; p = 0.721. Multivariable models confirmed these findings and demonstrated frailer patients had longer LOS and more readmissions. Currently, most UK renal transplant centres deliver a form of peri-operative ERAS care, indicating broad adoption of ERAS principles. Consequently, a formal ERAS protocol is not associated with decreased complications, LOS or readmissions. Efforts to improve outcomes should focus on prehabilitation of at-risk groups on the waiting list.

## Graphical Abstract

**Figure:**
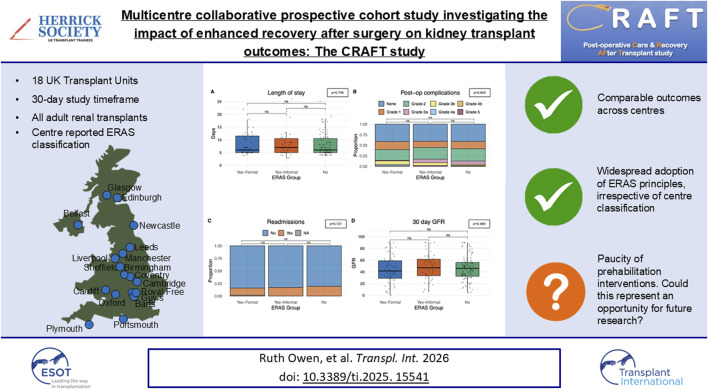

## Introduction

Kidney transplantation remains the optimal treatment for patients with end-stage kidney disease (ESKD), offering superior survival and quality of life compared to dialysis [1]. However, despite advancements in surgical techniques and immunosuppression, post-transplant complications, delayed graft function (DGF), and prolonged hospital stays continue to present significant challenges [2].

Enhanced Recovery After Surgery (ERAS) protocols, originally developed in colorectal surgery [3], have been increasingly adopted across multiple other surgical disciplines, demonstrating improvements in post-operative recovery, reduced complications, and shorter hospital stays [4]. The application of ERAS principles to kidney transplantation represents a promising strategy to optimize perioperative care and improve patient outcomes.

Broadly, ERAS protocols offer a suite of pre, intra and post operative goals or interventions, to guide surgical patient management, however local implementation of various aspects may differ. Preoperative measures focus on patient education, prehabilitation exercises, nutritional optimization, annual reviews on the waiting list, blood pressure management, smoking cessation and avoidance of prolonged fasting [5]. Intraoperatively, goal-direct fluid delivery, minimising the use of surgical drains, optimal anaesthetic protocols and opioid-sparing analgesia are emphasized to minimize physiological stress [6, 7]. Postoperative strategies prioritize early mobilization, multimodal pain management, and early oral intake to expedite functional recovery and reduce complications [8]. Evidence from non-transplant surgical specialties suggests that ERAS implementation leads to significant reductions in hospital length of stay, morbidity, and healthcare costs [9], but data on UK kidney transplantation remain limited to single respondent surveys [10], guidelines [11] or reviews.

Recent studies suggest that components of ERAS, such as restrictive fluid management and multimodal analgesia, may positively influence kidney transplant outcomes by reducing the incidence of DGF and improving early graft function [12, 13]. However, the efficacy and safety of a standardized ERAS protocol in this patient population have not been comprehensively evaluated prospectively across multiple centres. The effects of ERAS have been reviewed in a single centre setting, first by the Sheffield group [14] and included patient education and discharge planning (commenced on admission), carbohydrate loading, goal-directed fluid therapy, early oral intake post-operatively, early catheter removal (∼day 4), early drain removal and early mobilisation. Their results suggested a shorter length of stay (LOS) of 5 days (range 3–9 days), compared with a median LOS of 7 days (range 5–30 days) prior to the ERAS programme being implemented. A similar study was published by the Belfast group in 2021 which also showed a decreased LOS after implementation of their ERAS protocol [15]. Given the complexity of the kidney transplant recipient population, incorporating ERAS principles requires careful including consideration of immunosuppression regimens, fluid balance management, and recipient comorbidities [7, 8].

Using prospectively collected, real world data, this study aims to investigate the impact of ERAS implementation on kidney transplant outcomes, including length of hospital stay, postoperative complications, and readmission rates across multiple UK transplant centres. We compare kidney transplant recipients managed with and without an ERAS protocols, in order to determine whether this structured perioperative approach can enhance recovery and optimize transplantation outcomes. Understanding the role of ERAS in kidney transplantation may lead to standardized protocols that improve patient care, reduce healthcare costs, and enhance long-term allograft function.

## Patients and Methods

The CRAFT study was a multicentre prospective cohort study investigating the impact of ERAS on kidney transplant outcomes in the UK. Consecutive adult recipients undergoing live or deceased donor kidney transplantation at participating centres over a defined recruitment period were included. Paediatric recipients and those receiving multi-organ transplants (e.g., simultaneous pancreas kidney) were excluded.

Patients were categorized into *formal* ERAS, *informal* ERAS and *non*-ERAS centres based on a survey of the centre-reported care pathway collected prior to the data collection period. *Formal* ERAS centres were defined as those where an official ERAS protocol was in place which included preoperative optimization, intraoperative fluid and analgesic management, and postoperative recovery strategies that the department adhered to. Centres were classified as *informal* ERAS centres if they delivered ERAS-type care that was not protocolised but widely implemented and considered to be ERAS-like care by the department, and non-ERAS centres followed their standard surgical protocols. The non-ERAS centres served as the comparator group.

Data were collected prospectively for 30 days post-transplant using the Research Electronic Data Capture (REDCap™) system. The primary outcome was length of hospital stay (LOS). Secondary outcomes included the incidence of Clavien-Dindo grade ≥3 complications, graft function, and 30-day readmission rates.

This study was conducted as a national service evaluation project and local audit and research governance approvals were obtained for all participating centres by the responsible principal investigators. No changes to clinical care nor patient-identifiable data were stored in the REDCap™ system, and all data were anonymized before analysis.

### Statistical Analysis

Continuous data were summarized as medians with interquartile ranges, while categorical data were presented as counts and percentages. Differences between groups for continuous outcomes were assessed using the unpaired non-parametric Kruskal-Wallis test. For categorical data, the chi-squared test was used, and Fisher’s exact test was applied for groups with small sample sizes. Post hoc pairwise comparisons were conducted to identify which groups differed from one another. Wilcoxon rank-sum tests were used for continuous outcomes, and Chi-squared tests were used for categorical outcomes. To assess the impact of ERAS protocols on length of stay multivariable Cox regression models were used with a frailty term (random effect) for transplant centre. For this analysis each centre was considered a single cluster, even if the use of ERAS protocols varied at the patient-level. This hierarchical strategy accounts for the clustered nature of the data, whilst allowing adjustment for patient-level variables (including recipient, transplant and donor factors). For these analyses, LOS was treated as a time-to-event variable, with discharge being the event (with higher hazard ratios indicating faster discharge). All analyses were performed in R version 4.2.1 (R Project for Statistical Computing).

## Results

### Centre Reported Protocols

Eighteen adult kidney transplant centres across the United Kingdom participated in the study, of a possible 24. Each hospital submitted a centre-reported perioperative care pathway questionnaire which detailed whether they had a formal ERAS programme and what the standard elements of pre-, intra-, and post-operative care included, Table 1. This allowed us to categorise the centres. One hospital had an informal ERAS programme when they began the CRAFT study data collection, however, during the study period initiated a separate trial which brought in a formal ERAS programme. As such this hospital is treated as two separate centres throughout our descriptive analyses (Centre E − *informal* – to represent the patients prior to the trial starting, and Centre E – *formal* to represent the patients after the trial started). Another hospital, Centre K had an *informal* ERAS programme for living donor kidneys but no ERAS programme for deceased donor kidneys recipients. This centre was also treated as two “separate centres” within our descriptive analyses to ensure the difference in ERAS protocoled care was accounted for. Of the 20 separate centres, n = 5 had a *formal* ERAS protocol, n = 7 considered themselves to have *informal* ERAS care and n = 8 had *no specific* ERAS programme.

**TABLE 1:** Site survey results. Centre-reported ERAS implementation.

Centre	ERAS for recipients of:		ERAS Care includes			Routine pre-operative care						Routine intra- and post- operative care				
	LD	DBD/DCD	Nurse or coordinator	Pre-op counselling	Patient support document	Annual review on waiting list	Rehab exercise programme	Carb loading drinks	Nutrison optimisation	Smoking cessation	Weight and BP optimisation	Itra-op goal directed fluids	Ureteric stenting	Time to removal of stent (weeks)	Method of removal	Placement of surgical drain
Formal ERAS Centre																
A	Yes	Yes	​	​	​	Yes	Yes	Yes	​	Yes	Yes	Yes	Yes	3	Flexible cystoscopy with LA in OPD	​
B	Yes	N/A	​	​	​	Yes	​	Yes	Yes	Yes	​	Yes	Yes	4		Yes
C	Yes	Yes	​	​	​	Yes	​	​	​	Yes	​	​	Yes	6		​
D	Yes	Yes	Yes	Yes	Yes	Yes	Yes	​	​	Yes	Yes	Yes	Yes	2		Yes
E-formal	Yes	Yes	​	​	​	Yes	​	​	​	​	​	Yes	Yes	3		Yes
Total	100%	100%	20%	20%	20%	100%	​	​	​	​	​	80%	100%	x̄ = 3.6	​	60%
Informal ERAS Centre																
E-informal	Yes	Yes	​	​	​	Yes	​	​	​	​	​	Yes	Yes	3	Flexible cystoscopy with LA in OPD	Yes
F	Yes	Yes	​	​	​	Yes	​	​	​	​	​	​	Yes	6		Yes
G	Yes	Yes	​	​	​	​	​	Yes	​	​	Yes	Yes	Yes	2		​
H	Yes	Yes	​	​	​	​	​	​	​	​	​	​	Yes	6		​
I	Yes	Yes	​	​	​	Yes	​	​	Yes	Yes	Yes	Yes	Yes	6		Yes
J	Yes	Yes	​	​	​	​	​	​	​	​	​	​	Yes	2		​
K-LD	Yes	N/A	​	​	​	Yes	Yes	​	Yes	Yes	Yes		Yes	6		​
Total	100%	100%	0%	0%	0%	57%	14%	14%	29%	29%	43%	43%	100%	x̄ = 4.4	​	43%
Non-ERAS Centre																
K-DBD/DCD	N/A	No	​	​	​	Yes	Yes		Yes	Yes	Yes	​	Yes	6	Flexible cystoscopy with LA in OPD	​
L	No	No	​	​	​	Yes	​	​	​	​	​	​	Yes	6		​
M	No	No	​	​	​	Yes	​	​	Yes	​	Yes	​	Yes	6		​
N	No	No	​	​	​	​	​	​	​	​	​	​	Yes	6		Yes
O	No	No	​	​	​	​	​	​	​	​	​	​	Yes	2		​
P	No	No	​	​	​	Yes	​	​	​	​	​	​	Yes	3		Yes
Q	No	No	​	​	​	​	​	​	​	​	Yes	​	Yes	4		Yes
R	No	No	​	​	​	Yes	​	​	​	​	​	​	Yes	4		Yes
Total	0%	0%	0%	0%	0%	63%	13%	0%	25%	13%	38%	0%	100%	x̄ = 4.6		50%

Breakdown of units that considered themselves to have a formal ERAS protocol, and informal protocol or no protocol. Detailed information about the standard of care provided in the pre-operative, intra-operative and post-operative period. Abbreviations: BP – Blood Pressure DBD – Donation after Brainstem Death; DCD – Donation after Circulatory Death; ERAS – Enhanced Recovery After Surgery; LA- Local Anaesthetic; LD – Living Donor; OPD – Outpatient Department x͂ - mean.

213 transplants took place across the 20 centres during the 30-day study timeframe (15th January – 15th February 2024). Donor and recipient demographics were compared between centres that defined themselves as a formal ERAS centre, an informal ERAS centre a non-ERAS centre. Data completeness for this study was 99.8% and so a missing value analysis was not undertaken.

### Donor and Recipient Demographics

Donor demographics were comparable between groups with regards to donor sex, donor age, donor type (live/DBD/DCD), UK Donor Risk Index [16], HLA mismatch, and hypothermic machine perfusion. Warm ischaemic time (WIT) and cold ischaemic time (CIT) were also similar between the two groups.

Live donor transplants accounted for 34.9% (n = 37) or transplants performed in *formal* ERAS centres, 46.8% (n = 22) of transplants performed in an *informal* ERAS centre and 30.1% (n = 31) transplants performed in centres with *no* ERAS programme. The UK donor risk index (UKDRI) was calculated for all donors. There was comparable donor risk grafts utilised by *formal* ERAS centres (Median 1.4, IQR 0.7–1.6), when compared to *informal* centres (Median 1.4, IQR 1.1–1.7) and *non*-ERAS centres (Median 1.2, IQR 1.0–1.6), p = 0.615, Table 2. There were also a comparable number of donors having a renal transplant pre-emptively (defined as prior to the start of dialysis) in centres with a *formal* ERAS programme (n = 11, 17.5%), compared with *informal* centres (n = 10, 21.3%) and *non-*ERAS centres (n = 17, 16.5%). CIT was also comparable between those with a formal, informal or no ERAS programme, p = 0.213, Table 2.

**TABLE 2:** Donor Demographics. *Data shown as number + percentage.*

Donor Demographics	Variable	ERAS – Formal	ERAS – Informal	No ERAS	Total	p
Total	N (%)	63 (29.6)	47 (22.1)	103 (48.4)	213	​
Donor age	Median (IQR)	54.0 (36.5 to 63.5)	51.0 (39.0 to 62.5)	53.0 (42.0 to 62.0)	53.0 (39.0 to 63.0)	0.779
Donor sex	F	26 (41.3)	19 (40.4)	42 (40.8)	87 (40.8)	0.996
Donor type	Live	22 (34.9)	22 (46.8)	31 (30.1)	75 (35.2)	0.206
​	DBD	22 (34.9)	16 (34.0)	34 (33.0)	72 (33.8)	​
​	DCD	19 (30.2)	9 (19.1)	38 (36.9)	66 (31.0)	​
UKDRI	Median (IQR)	1.4 (0.7 to 1.6)	1.4 (1.1 to 1.7)	1.2 (1.0 to 1.6)	1.3 (0.9 to 1.6)	0.615
Terminal eGFR	Median (IQR)	90.0 (72.0 to 90.0)	83.0 (62.0 to 90.0)	90.0 (78.8 to 90.0)	90.0 (75.0 to 90.0)	0.040
Cold ischaemic time (minutes)	Median (IQR)	649.0 (301.0 to 963.0)	386.0 (234.5 to 834.0)	631.0 (317.5 to 927.0)	615.0 (249.0 to 902.0)	0.213
Warm ischaemic time (minutes)	Median (IQR)	46.0 (34.0 to 87.8)	22.5 (19.0 to 28.2)	38.5 (22.2 to 185.0)	38.5 (23.0 to 178.5)	0.097
HLA mismatch - DR	2	9 (14.3)	8 (17.0)	17 (16.5)	34 (16.0)	0.955
NRP	Yes	6 (9.5)	0 (0.0)	13 (12.6)	19 (8.9)	0.041
Hypothermic Machine perfusion	Yes	0 (0.0)	0 (0.0)	3 (2.9)	3 (1.4)	0.211

DBD – Donation after Brainstem Death, DCD - Donation after Circulatory Death. UKDRI – UK donor risk index. HLA – Human Leucocyte antigen, NRP- Normothermic Regional Perfusion.

Donor Terminal eGFR was statistically significantly lower (p = 0.040) in centres with an *informal* ERAS programme when compared to those with a *formal* programme and *no* programme at all, Table 2. NRP was also noted to be statistically significantly less likely to be utilised in the *formal* ERAS programme (p = 0.041).

There was no significant difference in any other recipient demographics between comparator groups. This included analysis of age, sex, body mass index (BMI - categorised by the WHO classification) [17], WHO performance status, number of previous transplants, urological pathology, anatomy, and immunosuppression regime, Table 3. Indicating that the groups were well matched.

**TABLE 3:** Recipient Demographics. *Data shown as number + percentage.*

Recipient Demographics	Variable	ERAS – Formal	ERAS – Informal	No ERAS	Total	p
Total	N (%)	63 (29.6)	47 (22.1)	103 (48.4)	213	​
Age	Median (IQR)	53.0 (43.5 to 60.0)	51.0 (36.0 to 62.0)	54.0 (40.5 to 61.0)	54.0 (40.0 to 60.0)	0.761
Sex	F	26 (41.3)	11 (23.4)	35 (34.0)	72 (33.8)	0.146
	M	37 (58.7)	36 (76.6)	68 (66.0)	141 (66.2)	​
BMI	Median (IQR)	27.9 (23.3 to 31.1)	26.1 (23.3 to 30.3)	27.0 (24.2 to 30.6)	26.7 (24.0 to 30.8)	0.457
WHO performance status	0	41 (65.1)	30 (63.8)	59 (57.3)	130 (61.0)	0.685
Pre-emptive transplants	None	11 (17.5)	10 (21.3)	17 (16.5)	38 (17.8)	0.505
Previous kidney transplants	None	54 (85.7)	40 (85.1)	87 (84.5)	181 (85.0)	0.897
Urological pathology	Yes	3 (4.8)	3 (6.4)	8 (7.8)	14 (6.6)	0.749
Standard anatomy	Yes	42 (66.7)	37 (78.7)	70 (68.0)	149 (70.0)	0.303
Immuno-suppression	Augmented	6 (9.5)	10 (21.3)	6 (5.8)	22 (10.3)	0.015

BMI- Body Mass Index. Urological pathology. Those with augmented immunosuppression relates to any immunosuppression protocol beyond that of standard immunosuppression.

### Intraoperative Patient Management

Prospective data was collected on intra- and peri-operative management to better understand how care was actually being delivered, alongside how centres had reported that they delivered care in the site survey.

**FIGURE 1:**
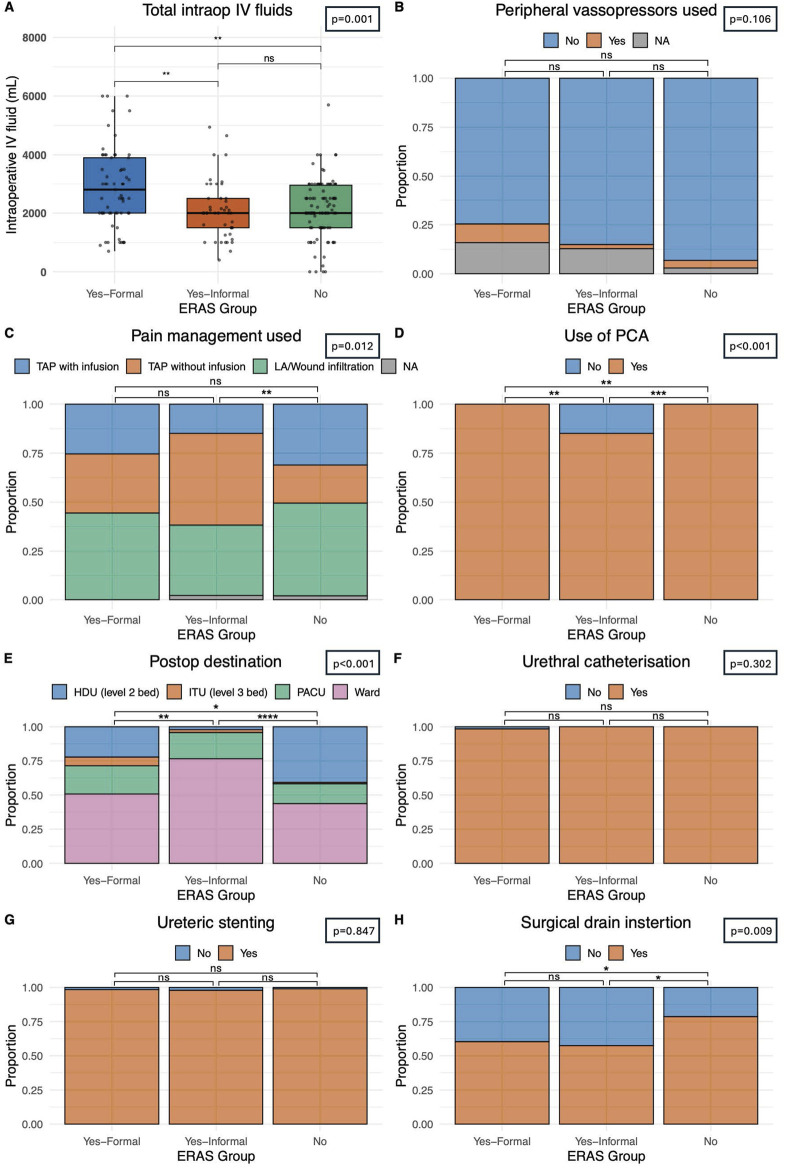
Delivery of perioperative care. **(A)** Intraoperative intravenous fluid usage in mL. **(B)** Intraoperative peripheral vasopressor usage. **(C)** Intraoperative pain management strategy including Transversus Abdominis Plan (TAP) block with or without infusion of local anaesthetic or local anaesthetic only. **(D)** Use of patient-controlled analgesia technique post-operatively. **(E)** Patient destination post operatively, including high dependency unit (HDU – level 2 care), intensive treatment unit (ITU – level 3 care), a prolonged stay within a post-anaesthetic care unit (PACU) or immediate ward-based care. **(F)** Incidence of urethral catheterisation pre- or intra-operatively. **(G)** Incidence of intraoperative ureteric stenting. **(H)** Incidence of surgical drain insertion. Bars indicate p-values from pairwise comparisons (Wilcoxon rank-sum test for continuous, and Chi-squared test for categorical variables). Significance levels: *p ≤ 0.05, **p ≤ 0.01, ***p ≤ 0.001, ****p ≤ 0.0001.

Volume of intraoperative fluid provided was compared between our groups. Centres with a *formal* ERAS protocol gave statistically significantly (p < 0.001) large fluid volumes (2,800 mL IQR: 2,000–3,900) when compared with *informal* ERAS centres (2,000 mL IQR: 1,500–2,950) and *non-*ERAS centres (2,000 mL IQR: 1,500–2,950), Figure 1A. Most patients (55.9%, n = 116) received TAP block analgesia with or without continuous infusion, intraoperatively. Patients in a centre with *no* ERAS programme were statistically significantly less likely to use a TAP block than those with a *formal/informal* programme (p = 0.012) Figure 1C. Patient controlled analgesia was utilised in 96.7% of transplants (n = 206). Of the seven patients who did not receive a PCA they were all within a single *informal* ERAS centre which was statistically significant (p < 0.0001), Figure 1D. Patients who received *formal* ERAS care were statistically significantly more likely to go back to the ward post-operatively (50.8%, n = 32), as were those who underwent *informal* ERAS care (76.6%, n = 36) when compared to those without an ERAS programme (43.7%, n = 45), p < 0.0001, Figure 1E where they were more likely to go to the high dependency unit. Those who underwent *formal or informal* ERAS care were also statistically significantly less likely to have a surgical drain inserted (p = 0.009). 60.3% of *formal* ERAS patient (n = 38) had a drain inserted, 57.4% (n = 27) in an *informal* centre and 78.6% (n = 81) in centres with *no* ERAS programme, Figure 1H. Comparable rates of peripheral vasopressor use (p = 0.106), Figure 1B, urethral catheterisation (p = 0.302), Figure 1F, and ureteric stenting (p = 0.847), Figure 1G, was seen in all centres.

### Post-Operative Management of Drains and Lines

**FIGURE 2:**
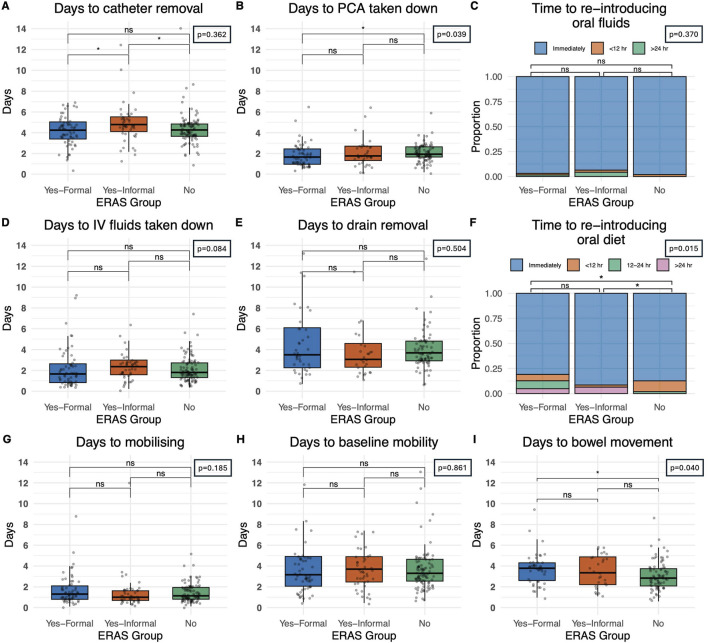
Impact of perioperative care approach on post-operative course. **(A)** Time until catheter removal, measured in days. **(B)** Time until patient controlled analgesia (PCA) taken down in recipients who were given a PCA. **(C)** Time until oral fluids reintroduced categorized as immediately post-operative, within 12 hours and greater than 24 h **(D)** Time until intravenous fluids taken down in days **(E)** Time until drain removal, measured in days **(F)** Time until re-introduction of oral diet categorized as immediately post-operatively, within 12 h, 12–24 h and greater than 24 h. **(G)** Time until the patient first mobilises, measured in days post-operatively **(H)** Time until patient returned to baseline mobility, measured in days **(I)** Days until first documented bowel movement, measured in days. Bars indicate p-values from pairwise comparisons (Wilcoxon rank-sum test for continuous, and Chi-squared test for categorical variables). Significance levels: *p ≤ 0.05, **p ≤ 0.01, ***p ≤ 0.001, ****p ≤ 0.0001.

Patients in centres with a *formal* ERAS protocol had earlier catheter removal (4.3 days IQR: 3.4–5.1) than those with an *informal* programme (4.8 days IQR: 4.1–5.5) and comparable catheter removal to centres with *no-*ERAS (4.3 days IQR: 3.7–4.9) protocol, Figure 2A (p = 0.036). Oral diet was introduced at a later stage in recipients who underwent a *formal* ERAS protocol (12.7%, at 24 h or more) when compared to centres with an *informal* programme (6.4%) or *no* ERAS programme (0%) (p = 0.015), Figure 2F. Centres with *no* ERAS protocol had a statistically significant shorter period of time post-operatively until documentation of bowel movement (2.8 days, IQR: 2.1–3.7), when compared to *formal* centres (3.8 days, IQR:2.6–4.2) and *informal* centres (3.3 days, IQR:2.2–4.9) (p = 0.040) Figure 2I. Patients in a *formal* ERAS centre had their PCA taken down earlier (1.7 days IQR: 0.8–2.6) when compared to centres with *informal* programmes (1.8 days IQR:1.3–2.7) and *non-*ERAS centres (1.9 days IQR:1.7–2.7), this reached statistical significance but is unlikely to be clinically significant given the actual values (p = 0.036), Figure 2B. The time from operation to re-introducing oral fluids (p = 0.370) Figure 2C, and IV fluids being taken down (p = 0.084), Figure 2D was comparable. Days until drain removal was also comparable (p = 0.504), and on average centres were removing drains after 3.6 days, Figure 2E. There were comparable outcomes when analysing the number of days from operation to mobilisation, those in a *formal* ERAS program mobilised at 1.3 days (IQR: 0.8–2.1), an *informal* programme 1.0 days (IQR: 0.7–1.6) and *no* ERAS programme 1.1 days (IQR: 0.8–1.9) (p = 0.185), Figure 2G. They also returned to baseline mobility at comparable timeframes, 3.2 days for patients in a *formal* ERAS programme (IQR: 2.1–4.9), 3.7 days in the *informal* programme (IQR: 2.5–4.9) and 3.3 days for patients with *no* ERAS programme (IQR: 2.5–4.6), (p = 0.861), Figure 2H. Patients in the non-ERAS centres opened their bowels sooner than patients in formal ERAS centres (p = 0.040), Figure 2I.

### Post-Operative Outcomes

**FIGURE 3:**
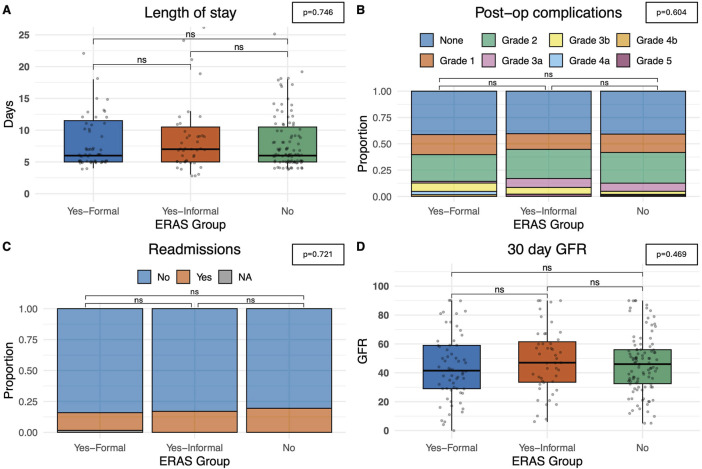
Postoperative outcomes. **(A)** Length of stay, measured in days from admission. **(B)** Total complications noted, categorised by Clavien-Dindo grading score. **(C)** Number of readmissions within a 30-day time-period. **(D)** eGFR at 30 days. Bars indicate p-values from pairwise comparisons (Wilcoxon rank-sum test for continuous, and Chi-squared test for categorical variables). Significance levels: *p ≤ 0.05, **p ≤ 0.01, ***p ≤ 0.001, ****p ≤ 0.0001.

The primary outcome measured in this study was the length of stay (days since transplant) and no statistically significant differences on univariate analysis were observed between *formal* ERAS centres, or *informal* ERAS centres when compared with *non-*ERAS units (p = 0.746). The average length of stay in centres with a *formal* ERAS programme was 6.0 days (5.0–11.5), 7.0 days (5.0–10.5) in centres with *informal* ERAS and 6.0 (5.0–10.5) days in centres without an ERAS protocol, Figure 3A. A comparable rate of post-operative complications (categorised using the Clavien-Dindo Grading system) (p = 0.604), were observed. 14.3% (n = 9) patients in the *formal* ERAS programme had a Grade 3 or higher Clavien-Dindo complication, comparable to the 17% (n = 8) in the *informal* ERAS programme and 12% (n = 12) in centres with *no* ERAS programme, Figure 3B.

**FIGURE 4:**
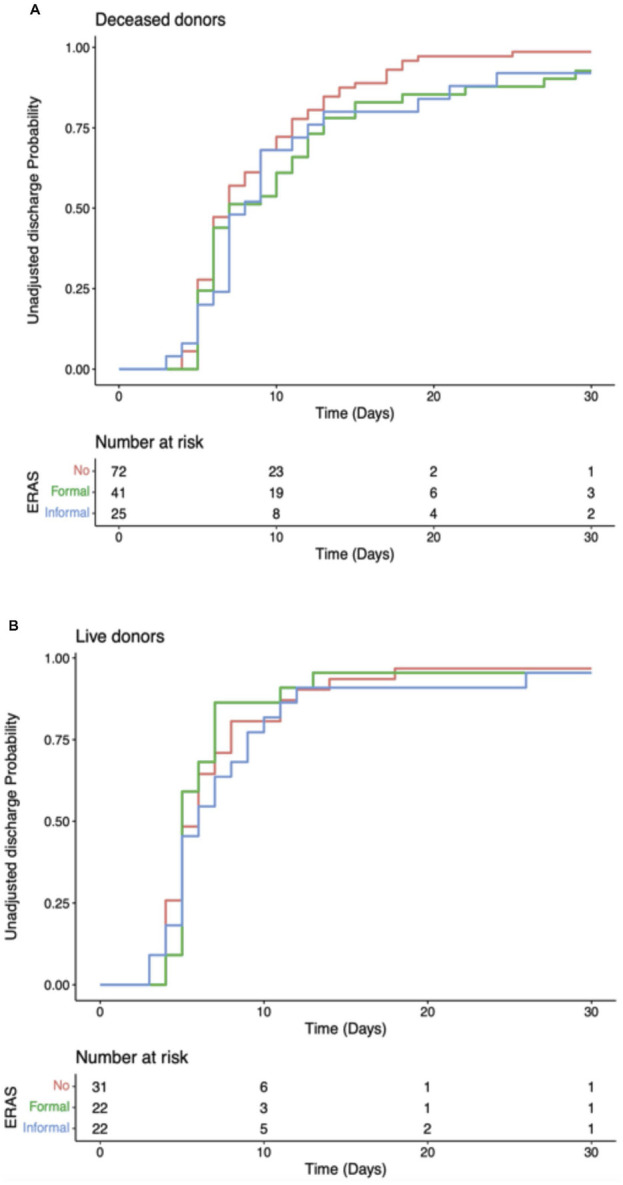
Cumulative unadjusted discharge probability after transplantation, stratified by transplant unit ERAS group. **(A)** Recipients of deceased donor kidney. **(B)** Recipients of live donor kidney.

The recipients also had comparable rates of readmission at 30 days. In centres with a *formal* ERAS programme 14.3% (n = 9) patients were readmitted, 17% (n = 8) in centres with *informal* ERAS programmes and 19.4% (n = 20) in centres without an ERAS programme (p = 0.721), Figure 3C. Median eGFR 30 days post-operatively was also comparable. In f*ormal* ERAS centres median 30 days eGFR was 41.5 mL/min/1.73 m^2^ (IQR: 29.0–59.0), 47.0 mL/min/1.73 m^2^ in *informal* centres (IQR: 33.5–61.5) and 46.0 mL/min/1.73 m^2^ (IQR: 32.5–56.0), (p = 0.469), Figure 3D. Univariate analyses were also performed delineating recipients who received a graft from a deceased donor (DBD/DCD graft) and those who received a graft from a living donor. These models showed comparable time until discharge irrespective of whether the centre described themselves as having a *formal* ERAS protocol, and *informal* protocol or *no* ERAS protocol. This trend was similar in both deceased and live donors (Figure 4).

### Multivariable Analysis of Impact of ERAS on Length of Stay

A multivariable model was created to analyse the impact of ERAS protocols within the context of other recipient and donor factors including type of transplant (DBD/DCD/Live Donor) as well as patient frailty (based on the WHO performance status), Table 4. In the multivariable hierarchical Cox regression model, ERAS status was statistically significantly associated with length of stay (Wald p = 0.030). This was demonstrated by a lower HR for discharge (formal ERAS aHR = 0.753, 0.543–1.045, p = 0.090 and informal ERAS aHR = 0.628, 0.435–0.906, p = 0.013; Table 4); adjusted length of stay was longer in the centres with ERAS programmes compared to those without ERAS programmes. The multivariable models also demonstrated patients with WHO performance status ≥1 (frailer patients) had longer LOS, Table 4. A sensitivity analysis adjusting for pre-emptive transplant as a confounder was performed which demonstrated similar results. A further sensitivity analysis was performed excluding the two centres which used a mixture of ERAS and no ERAS; results were in keeping with the model in Table 4. Finally, we repeated the model shown in Table 4, instead categorising ERAS status into “formal ERAS” versus “no formal ERAS” (combining the “no ERAS” and “informal ERAS” groups). There was no significant difference in length of stay between “formal ERAS” and “no formal ERAS” (aHR = 0.888, 0.647–1.219, 0.462).

**TABLE 4:** Multivariable hierarchical Cox regression model for length of stay, with random effect term for transplant centre.

Variable	HR (95%CI)	P value
ERAS - none	1	​
ERAS - formal	0.753 (0.543–1.045)	0.090
ERAS - informal	0.628 (0.435–0.906)	0.013
Live	1	​
DBD	1.051 (0.748–1.476)	0.774
DCD	0.462 (0.326–0.656)	0.000
WHO performance status 1	0.586 (0.423–0.811)	0.001
WHO performance status >1	0.544 (0.328–0.904)	0.019
Random effect for transplant centre	Random effect	0.916

Length of hospital stay is modelled as time to discharge, and therefore hazard ratios lower than 1 represent prolonged hospital stay. ERAS – Enhanced Recovery After Surgery; DBD – donation after brainstem death; DCD – donation after circulatory death.

## Discussion

ERAS was conceptualised within colorectal surgery and has since been adopted across various surgical disciplines, with specialty-specific adaptations to complement the demographic of patients and the operations being performed. In this study, across multiple UK kidney transplant centres, we demonstrate that the uptake of a specific ERAS protocol in renal transplant has been variable. However, despite this, the delivery of perioperative care was very similar across centres irrespective of how the centres categorised themselves with all centres tending towards ERAS-style care. From the study we noted that regardless of a centres classification, the cautious use of IV fluids, vasopressors, patient controlled analgesia, avoidance of epidurals and strategic drain insertion were commonplace. This likely represents a wider culture change across all surgical specialties as ERAS principles have become embedded in standard UK surgical practice. This lack of difference in the real-world delivery of perioperative care may also explain the similar length of stay between cohorts.

Of note, pre-operative ERAS style care was found to be less well embedded within practice. Few centres offered nutritional support, or pre-operative carbohydrate loading drinks and less than half of centres offered weight advice and blood pressure optimisation. Exercise programmes were also sparsely available. Those with formal ERAS protocols had a greater propensity for smoking cessation programmes than centres without. These differences may reflect the relatively unpredictable nature of deceased donor transplantation.

The renal transplant recipient population are generally more comorbid than those undergoing other elective general surgery, in part as a consequence of end stage renal disease, and therefore organ support in the form of chronic dialysis dependence. Additionally, there are changing demographics within the kidney recipient population, over time–tending towards greater numbers of older, co-morbid transplant recipients [18], as the population ages. This study found that recipients with a worse WHO performance status had an associated increased length of stay, as would be expected. As such this would suggest that the ERAS elements of pre-operative optimisation and prehabilitation which are less well implemented across the board, may aide improvements in a patient’s functional status and may represent another target to improve outcomes.

Donor and recipient demographics were largely comparable between centres with a *formal* ERAS programme, an *informal* ERAS programme and *no* ERAS programme, thus demonstrating that our cohorts were well matched for comparison with no one group having a high proportion of pre-emptive live donor transplants which would artificially skew length of stay data. There were two differences within the donor demographic group which reached statistical significance, donor terminal eGFR and the use of NRP. For donor terminal eGFR centres with an informal ERAS programme accepted grafts from donors with a statistically significantly poorer terminal eGFR (p = 0.040). Whilst statistically significantly different we do not think the difference in eGFR noted would be clinically significant when looking at the absolute values. Terminal eGFR is also a single value and therefore does not discriminate between an acute injury to the kidney that may be recoverable, precipitated by the mechanism of death in the donor, versus chronic kidney disease. With regards to the use of normothermic regional perfusion (NRP), this is an emerging technique with limited centres of expertise and no centralised funding. Grafts from donors who underwent NRP were statistically significantly less likely to be accepted by programmes with an *informal* ERAS programme. We believe this is likely coincidental secondary to the geography of the retrieval units routinely performing NRP rather than directly related to the ERAS programme. Recipient demographics were similar between all centres which provides confidence when comparing our primary and secondary outcomes.

This study provides a unique snapshot of real-world perioperative care delivery in kidney transplantation across multiple centres. Importantly, our findings indicate that whilst only 27% of centres would describe themselves to have a formal ERAS programme, most UK renal transplant centres are delivering perioperative ERAS-type care. The average length of stay for all patients in the study was 6 days. A prior study using data from 2020 showed an average of 10 days stay, which could be improved to 5–7 days in units with an active ERAS programme [10]. This suggests that the principles of ERAS have been widely adopted into routine clinical practice, which is reflected in the improved lengths of stay, and a broader trend towards optimizing management in kidney transplantation.

Despite the widespread adoption of ERAS-type care, our univariate unadjusted analysis found no significant association between the implementation of a *formal* ERAS protocol and reduced complications, length of stay (LOS), or readmissions. However, the adjusted analysis did demonstrate an increased length of stay in the *informal* ERAS cohort. This could be explained by the fact that centres without ERAS protocols may already operate with a relatively short LOS, hence there is no need to introduce an ERAS protocol, limiting the potential for further reductions. Notably, the median length of stay across the different cohorts was relatively acceptable at 6 days. This is a considerable reduction from 20 years ago when often renal transplant recipients would often have significantly longer lengths of stay, with over 20% of US recipients staying in hospital for more than 2 weeks post-transplant [19]. This highlights how the culture change has become embedded within all renal transplant centres to encourage early discharge.

These findings also highlight the paucity of preoperative optimization efforts which may represent an area for improvement. Rather than solely emphasizing intraoperative ERAS implementation, we suggest future strategies should prioritize addressing patient-related factors that impact recovery. Specifically, prehabilitation and frailty management for at-risk patients on the transplant waiting list could provide a more effective means of improving perioperative outcomes. Prehabilitation has been described as a process where a patient’s functional capacity is enhanced prior to surgery in preparation for the known upcoming stressor which is surgery [20].

There are four main aspects of prehabilitation which include: medical optimisation, nutritional support, increasing physical exercise, and psychological support. Medical optimisation focusses on smoking cessation to improve post operative wound healing [21] and weight management – for both obese and underweight patients, both of whom are at risk of malnutrition [22]. Patient malnutrition is associated with increased length of stay, infection, increased readmissions and mortality [23] and strategies to improve nutrition, including preoperative carbohydrate loading drinks and a high protein diet in the weeks prior to surgery to reduce insulin resistance and improve immune responses [24]. Physical exercise is well documented to have improved benefits post-surgery, including decreasing length of stay, but is notoriously challenging with regards to patient uptake [25]. In transplantation, the unpredictable and variable timing from listing to transplant makes the delivery of prehabilitation difficult as patients need to be able to maintain the gains made throughout a potentially extended waiting period for an organ offer to become available. This may be more achievable in live donor transplantation as a planned elective operation and prompts the question should we be encouraging more of our comorbid and frail recipients to go for this approach alongside targeted prehabilitation? This could be combined with the known benefits of pre-emptive transplantation to avoid the compounded effect of dialysis in this at-risk cohort [26].

### Study Limitations

The main limitation of our study is its observational nature, consequently assessments of causality cannot clearly be made. Additionally, we acknowledge that the follow up period, is limited: the study was actively recruiting for 30 days and had a further 30 days of follow up which is a relatively short time period. This was deliberately chosen to be pragmatic, as our contributors were trainees who often move centre during training. Despite this, we had well matched cohorts across 20 different centres with over 200 patients and for the majority of patients a 30 days follow up period is more than adequate to capture outcomes of interest in the early postoperative period that were of interest in this scenario e.g., length of stay, readmission and complications. The study was designed as a prospective service evaluation study which allowed for multiple centres to be involved due to the simpler registration and approval processes. Again, this was a pragmatic decision, as the real world costs associated with running a multicentre randomised control trial of an ERAS protocol would be prohibitive, not to mention challenging to ensure adherence. As such, this is the largest prospective study to date assessing the role of ERAS care in renal transplantation, which provides a clear snapshot of perioperative practice across the UK and potential insights for future improvements.

### Conclusion

In conclusion, while intraoperative ERAS principles have been widely integrated into routine kidney transplant care, formal ERAS protocols were not associated with significant improvements in post-transplant outcomes. This study found few centres offered prehabilitation strategies. Future efforts should focus on identifying high-risk patient populations and implementing prehabilitation strategies to enhance recovery and reduce complications. Further research is needed to explore how this would impact patient outcomes specifically within transplant patients and how we can achieve the same culture change for preoperative care that we have seen in the perioperative care setting to further optimize transplant outcomes effectively.

## Data Availability

Anonymized raw data supporting the conclusions of this article will be made available on reasonable request.

## Group Members of the CRAFT Study COLLABORATORS

Lauren Hackney, Damian McGrogan, Clara Tohill, Shauna McBride, Isobel Austin, Belfast City Hospital; Kayani Kayani, Dilan Dabare, Ugochukwu Okafor, Raja Rashid, Poppy Brown, Irena Radnaeva, Tariq Ghattas, Birmingham Queen Elizabeth Hospital; Ahmed Radwan, Harry VM Spiers, Catriona Walker, Paula Appleton, Cambridge Addenbrooke’s Hospital; Charlie Brown, Christopher Chalklin, Laszlo Szabo, Cardiff University Hospital of Wales; Farhan Ahmad, Hamza Ahmad, Keno Mentor, Coventry University Hospital; Katie L Connor, Rachel Thomas, Karen Clark, Neil McKane, Eksha Gupta, Edinburgh Royal Infirmary; Robert Pearson, Emma Aitken, Aldo Alonso-Becerra, Glasgow Queen Elizabeth University Hospital; Jessica Weemes, Adam Barlow, Sadia Tasleem, Sunil Daga, Melissa Bautista, Amirh Azhar, Leeds St James’ University Hospital; George Nita, Petra Goldsmith, Caitlin Jordan, Liverpool Royal University Hospital; Benedict Phillips, Hannah Maple, Ahmed Hussein, Yasaman Nikooiyan, Fayyad Jaradat, London – Guy’s Hospital; Zoja Milovanovic, Rebecca Matthews, Mohammad Ayaz Hossain, Fiona McCaig, Baven Blanderan, James Sweatman, London – The Royal Free Hospital; Christopher Seet, Muhammad Khurram, Ben Lindsey, Ismail Mohamed, Laura Clementoni, London – The Royal London Hospital Barts; Ruth Owen, Hussein Khambalia, Malcom Greenwood-Morgan, Orus Erum, Manchester Royal Infirmary; Emily Thompson, Aimen Amer, Carrie Scuffell, Tzer En Yap, Eleanor Kissane, Laura Kenny, Batool Almoosawi, Joe Dobbins, Sam Tingle, Jenny Nur, George Kourounis, Sarah Abdelbar, Newcastle Freeman Hospital; Hatem Sadik, Mohamed Aly M. El Shafei El Zawahry, Srikanth Reddy, Thomas Whitehead, Nasir Al-Karboolee, Muhammad Sheharyar Khan, Faraaz Khan, Oxford Churchill Hospital; Balaji Mahendran, Charlotte Hitchins, Sundar Muneeswaran, Haitham Alzoubi, Plymouth Derriford Hospital; Catherine Boffa, Abris Szentpali, Portsmouth Queen Alexandra Hospital; Bishow Bekhyat Karki, Yazine Marie, Ahmed Halwa, Sheffield Northern General Hospital.
